# Patients’ appraisals about a multicomponent intervention for fibromyalgia syndrome in primary care: a focus group study

**DOI:** 10.1080/17482631.2021.2005760

**Published:** 2021-11-29

**Authors:** Victoria Mailen Arfuch, AQ Gonçalves, Rosa Caballol Angelats, Carina Aguilar Martín, Noèlia Carrasco-Querol, Maria Cinta Sancho Sol, Gemma González Serra, Immaculada Fusté Anguera, Anna Berenguera

**Affiliations:** a Unitat de Suport a La Recerca Terres de l’Ebre, Institut Universitari d’Investigació En Atenció Primària Jordi Gol (Idiap Jordi Gol), Tortosa, Spain; b Department of Pediatrics, Obstetrics and Gynecology, and Preventive Medicine, Universitat Autònoma de Barcelona, Spain; c Insurance Medicine Division, Department of Neuroscience, Karolinska Institutet, Sweden; d Unitat Docent de Medicina de Família I Comunitària Tortosa-Terres de L‘Ebre, Institut Català de La Salut (Ics), Spain; e Centre d’Atenció Primària Baix Ebre Tortosa Est, Gerència Territorial de Terres de l’Ebre, Institut Català de La Salut (Ics), Tortosa, Spain; f Unitat d’Expertesa En Sindromes de Sensibilització Central Terres de l’Ebre, Gerència Territorial de Terres de l’Ebre, Institut Català de La Salut (Ics), Tortosa, Spain; g Unitat d’Avaluació, Direcció d’Atenció Primària Terres de l’Ebre, Gerència Territorial de Terres de l’Ebre, Institut Català de La Salut (Ics), Tortosa, Spain; h Centro de Salut Mental Adultos (Csma) de Fundació Pere Mata Terres de l’Ebre, Tortosa, Spain; i Servei de Rehabilitació I Medicina Física, Hospital Verge de La Cinta, Gerència Territorial de Terres de l’Ebre, Institut Català de La Salut (Ics), Tortosa, Spain; j Central Research Unit, Institut Universitari per a La Recerca a l’Atenció Primària de Salut Jordi Gol I Gurina (Idiapjgol), Barcelona, Spain; k Departament d’Infermeria, Universitat de Girona, Girona, Spain

**Keywords:** Fibromyalgia syndrome, primary care, multicomponent intervention, qualitative research, focus group discussions

## Abstract

**Purpose:**

To assess fibromyalgia patients’ experiences and appraisals about a multidisciplinary intervention programme, in Catalonia’s primary care, regarding its format and contents, benefits, and health impact in the short and long term.

**Method:**

Qualitative interpretative research design through hermeneutic phenomenology perspective. Two focus groups discussions were conducted in February and July 2020. The purposive heterogeneous sample included 19 fibromyalgia patients who attended a multicomponent programme. In addition, thematic analysis on the verbatims was performed.

**Results:**

Findings were organized into five main domains with an explanatory theme each. Overall, the informants valued the programme as a positive experience due to its holistic approach, health benefits, suffering relief, group effect, and fibromyalgia legitimacy promotion. Detected improvable aspects focused on extending the timeframe, including family members as beneficiaries, deepening the thematic contents, and getting regular access to this healthcare service. Furthermore, the intervention was considered feasible to be incorporated into usual clinical care.

**Conclusion:**

the programme fulfilled users’ expectations about results and procedure and showed promise as a treatment strategy to reinforce the usual practice. Our findings suggest a broad perspective on fibromyalgia patients’ suffering, which urges us to adjust the intervention programme to their real health needs.

## 1 Introduction

Fibromyalgia syndrome (FMS) remains medically unexplained (Alciati et al., 2021), for what its gold-standard treatment is debated, and there is scope for high-quality evidence (Mascarenhas et al., 2021). Being classified as a central sensitivity syndrome (Fleming & Volcheck, 2015), FMS significantly compromises patients’ quality of life (QOL) and functionality, leading to disability (Wuytack & Miller, 2011). Therefore, healthcare strategies need to provide biopsychological and multimodal treatment approaches in order to face FMS impact (Bair & Krebs, 2020).

Given FMS high prevalence among rheumatic illnesses, representing 2.45% in Spain (Cabo-Meseguer et al., 2017; Seoane-Mato et al., 2019), the usual clinical care (UCC) may not be sufficient to address patients’ suffering. In this context, primary care professionals, including nurses, physiotherapists, and psychologists, could strengthen the usual medical practice.

Clinically, FMS involves somatic, psychological, and social factors (Sarzi-Puttini et al., 2020). It is characterized by chronic widespread musculoskeletal pain associated with multiple unspecific symptoms such as insomnia, fatigue, depression and anxiety. As a result, patients’ social and work performance can be deteriorated leading to sickness absence and early retirement (Isomeri et al., 2020; Mas et al., 2008). Even though the evolution of its diagnostic criteria (F Wolfe et al., 1990, 2016; Frederick, 2010), both professionals and patients still struggle when searching for an accurate diagnosis and treatment approach (Galvez-s & Reyes, 2020).

Recent studies highlight the disruptive effect of the diagnosis experience and the treatment process on patients’ daily life (Ashe et al., 2017; Briones-Vozmediano et al., 2015; Taylor et al., 2016). This is especially true for middle-aged women, who are the most impacted population group showing a substantially higher rate than men (Marques et al., 2017; Mas et al., 2008; Villanueva et al., 2004). Even though this major sex difference is not fully understood, it could be explained by a diagnostic criteria bias (Häuser et al., 2019; Samulowitz et al., 2018; Frederick Wolfe et al., 2018). On the other hand, Martínez-Lavín (2021) proposes considering fibromyalgia as a stress-induced, sex-dimorphic neuropathic pain syndrome rather than a mental somatic symptom disorder, which may explain why it is more frequent in women due to the prevalence of this phenomenon in this population group.

According to the qualitative literature, the most frequent themes on patients’ accounts related to their living experience with FMS are poor functional performance, distress, lack of credibility, uncertainty, pain acceptance, poor sleep, and social stigma (Climent-Sanz et al., 2021; Johnson et al., 2006; LaChapelle et al., 2008; Quintner, 2020; Sim & Madden, 2008; Taylor et al., 2016)

Lempp et al. (2009) explain that the knowledge gap on FMS etiopathogenesis creates uncertainty about the best treatment option and foments FMS inauthenticity. In this scenario, patients’ voices reach a strategic value in the development of healthcare approaches.

The UCC for FMS in Spain is generally based on the administration of a medical record, the delivery of information about the condition, the prescription of pharmacological treatment, and the referral to specialists if necessary (Celaya et al., 2017; Gándara & Muñoz, 2017; Instituto Nacional de la Seguridad social, 2019). However, its purely biomedical approach seems limited as a healthcare strategy. Alternatively, non-pharmacological therapies such as physical activity, psychological therapy, and health education interventions have been demonstrated to improve symptom management and QOL (Aman et al., 2018; Baranowsky et al., 2009; Bernardy et al., 2013; Bush et al., 2013; García-Ríos et al., 2019; Luciano et al., 2014; Prabhakar et al., 2019; Sosa-Reina et al., 2017).

Furthermore, multicomponent interventions (MCI) have been proven beneficial in the short and long-term (Bourgault et al., 2015; Giusti et al., 2017; Jacobs et al., 2020; Martin et al., 2012; Ollevier et al., 2019; Saral et al., 2016) and recommended by international guidelines (Thieme et al., 2017). Nonetheless, this intervention approach is in its infancy in the Spanish public health sector.

Since 2016, accredited units specializing in Central Sensitivity Syndromes have provided multidisciplinary healthcare to FMS patients in Catalonia’s primary care centres and hospitals (Departament de Salut, Generalitat de Catalunya, 2017). As Stein and Miclescu (2013) explain, primary care is an appropriate setting for delivering treatment on chronic pain and supporting patients and their families with health education and coping skills.

To the best of our knowledge, qualitative research about the benefits of multidisciplinary treatments remains scarce (Bourgault et al., 2015; Miranda et al., 2016; Oliveira et al., 2019; Susmita Kashikar-Zuck et al., 2016). However, through a mixed-methods design, Bourgault et al. (2015) have evidenced that qualitative research is suitable for detecting patients’ global impression of change regarding pain management, functionality, and QOL.

According to the Medical Research Council in Implementation Science (NIH, 2018), communication with stakeholder groups is a valuable resource for assessing quality, acceptability, feasibility, and the contents of healthcare interventions. In addition, understanding participants’ treatment lived experience and its impact on daily life helps adapt the implemented practices to their true health needs and priorities (McMahon et al., 2012; Sim & Madden, 2008). Furthermore, focus group discussions (FGDs) are among the most popular data collection methods implemented in qualitative research (Gill et al., 2008) based on interpersonal interaction dynamics to extract information about the participants’ experiences and beliefs on a specific topic.

This study aims to assess patients’ experiences and appraisals about a complex intervention programme for FMS in primary care centres belonging to the Gerència Territorial Terres de L’Ebre of the Institut Català de la Salut, Spain. Precisely, this study intends to detect the improvable aspects of this programme, its barriers and facilitators, the adequacy of its elements (timeframe, setting, materials, group-based approach, among others), the quality of the therapeutic components, the relevance of the thematic content, its benefits on symptom control, and its impact on QOL in the short and long-term. The results are expected to tailor the intervention according to the patients’ actual health needs and available resources to strengthen the programme benefits and implementation. Additionally, this study may support and extend the results of a randomized clinical trial (RCT) linked to this project (ClinicalTrials.gov: NCT04049006) (Caballol Angelats et al., 2019). Finally, this study will allow the intervention programme’s standardization to be adjusted and replicable in other healthcare contexts and promote adherence.

## 2 Materials and methods

### 2.1 Design

Qualitative interpretative research was conducted following a hermeneutic phenomenological perspective (Dibley et al., 2020; Wilson, 2000). This design approach helps detect and interpret participants’ common meanings engaging what, why, and how questions in the intersubjective setting (Charmaz, 2008; Laverty, 2003; Tindall, 2009; Villegas, 1992).

### 2.2 Multicomponent intervention programme

The MCI implemented consists, in addition to the UCC, in a 12-week/2-hour session group-based programme combining: health education -including an introduction to multicomponent therapy, neurophysiology and pharmacology of pain, techniques of postural hygiene, nutrition, insomnia management, memory, and sexuality; physical exercise -focus on breathing and relaxation, stretching, strength/joint, and coordination exercises; and cognitive-behavioural therapy (CBT) -based on pain and attention management, learning to manage emotions, strategies for coping with difficulty, and pleasurable activities planning. It is delivered by a general practitioner, a physiotherapist, a psychologist, and each health centre’s head nurse.

This MCI programme aims to promote patients’ literacy and skills development for FMS management, enhance their physical status and reduce their emotional distress in order to overcome psychological difficulties. Further details about the thematic contents of each session and the research specifications were published in the study protocols of the RCT study and the qualitative research (Arfuch et al., 2020; Caballol Angelats et al., 2019).

### 2.3 Participants

All participants were recruited from the 11 primary care centres in the Gerència Territorial Terres de l’Ebre.

Purposive heterogenous sampling was implemented to reach maximum discursive variability considering gender, age, birth country, educational level, occupational situation, working status, and geographical area. According to the RCT data collection schedule, this sampling strategy divided the sample into two groups to assess the perception of benefits in the short and medium/long-term. As a result, the first FGD (FGD1) was performed in February 2020, including patients with ≥6 and 12 months of follow-up post-intervention. The second one (FGD2) was conducted in July 2020, including patients up to <6 months of follow-up.

The inclusion criteria involved a clinical diagnosis of FMS (International Classification of Diseases-10 codes: M79.0, M79.7), over 18-year-old, language skills in Catalan or Spanish, a phone number, a minimum of 75% attendance at the MCI programme (equal to or more than 9 out of 12 sessions), voluntary participation in the study and signed informed consent. On the other hand, the exclusion criteria included: active psychotic episode, intellectual impairment, severe depression and or personality disorder, auto/hyper-aggressive behaviour, psychoactive substances administration, not having met the minimum attendance, not signing the informed consent.

The recruitment was conducted three weeks in advance and via phone calls to those patients who met the inclusion criteria. They were provided with key information about the study goals and the date and place where the FGD would be carried out. In order to prevent absenteeism, two telephone contacts were made as reminders a week before the FGD date and the previous day.

**Figure 1.**
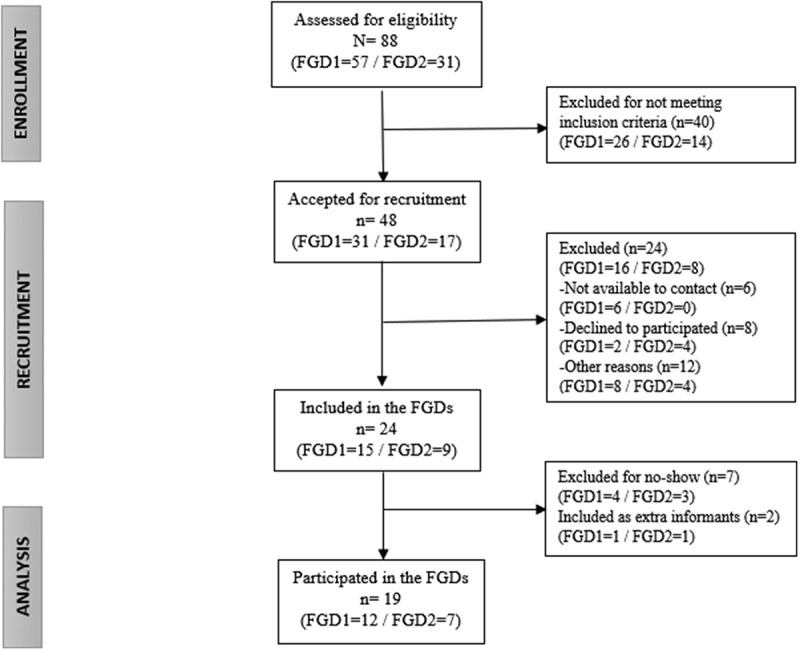
FGDs flow chart

As Figure 1 shows, from the 88 subjects (85 women/3 men) that participated in the MCI during the planned periods (FGD1 = 57; FGD2 = 31), 40 (39 women/1 man) were excluded for not meeting the inclusion criteria of 75% attendance. Consequently, 48 subjects (46 women/2 men) were included in the recruitment process, from which 24 were excluded, leaving 24 possible participants in the first lists. Finally, 19 informants participated in the FGDs (FGD1 = 12; FGD2 = 7) and were included in the thematic analysis. In addition, two members of this final sample were included as extra informants in the last call, although they had 66.7% of attendance in case of no-shows.

The study sample includes 63.2% of participants with a follow-up >6 months and 36.8% with <6 months. The sample comprises women as the only two men who met the inclusion criteria did not accept participating in the FGDs due to the COVID-19 outbreak. Furthermore, the participants belonged to 7 of the 11 primary care centres involved in the study. The attendance criterion was fulfilled with a total mean of 10.3 sessions attended (SD 1.2), representing a mean of 85.5% of participation and no substantial differences between focus groups. Table I shows a description of the study sample.

**Table I.** Study sample

	Total sample	FGD1 (> 6 months follow-up)	FGD2 (< 6 months follow-up)
	*N = 19*	*n = 12*	*n = 7*
	***Mean (SD) min-max***		
Age	61.8 (8.4) 46–79	59.4 (5.2) 53–71	65.8 (11.5) 46–79
Diagnostic evolution	7.9 (5.5) 1–19	7.3 (5.3) 2–16	9.1 (6.2) 1–19
Participation in the MI	10.3 (1.2) 8–12	10.4 (1.2) 8–12	10 (1.2) 8–11
	***N(%)***		
Country of birth			
Spain	18 (94.7)	12 (100)	6 (85.7)
Other	1 (5.3)	0	1 (14.3)
Marital status			
divorced	3 (15.8)	2 (16.7)	1 (14.3)
married	13 (68.4)	9 (75)	4 (57.1)
single	1 (5.3)	0	1 (14.3)
widow	2 (10.5)	1 (8.3)	1 (14.3)
Occupational situation			
employed	7 (36.8)	6 (50)	1 (14.3)
unemployed	1 (5.3)	1 (8.3)	0
disabled	2 (10.5)	2 (16.7)	0
homemakers	3 (15.8)	1 (8.3)	2 (28.6)
retired	6 (31.6)	2 (16.7)	4 (57.1)
Educational level			
primary	12 (63.2)	8 (66.7)	4 (57.1)
secondary	4 (21.1)	4 (33.3)	0
post-secondary	1 (5.3)	0	1 (14.3)
without studies	2 (10.5)	0	2 (28.6)
Work status			
hight professionals	1 (5.3)	0	1 (14.3)
intermediate professionals	2 (10.5)	2 (16.7)	0
skilled white-collar workers	6 (31.6)	6 (50)	0
skilled manual workers	0	0	0
manual labourers	10 (52.6)	4 (33.3)	6 (85.7)
SD = standard deviation			

Table I. Study sample.

Among the most remarkable characteristics of the sample, the informants’ mean age was 61.8 years, being the second FGD more mixed-age than the first one. FMS evolution was estimated based on the diagnostic year showing a mean of 7.9 years (SD 5.5), with approximately 2 points of difference between groups. Furthermore, 94.7% of the sample were born in Spain, and the most frequent marital status was married (68.4%) in both FGDs. Regarding the educational level, 73.7% of the total sample had no studies or just a complete primary education. In reference to the occupational situation, 36.8% of the sample were employed while 47.4% were unemployed, disabled, or retired; and only 15.8% were homemakers. However, 50% of the informants in FGD1 were employed, whereas in FGD2, it represented 14.3% as 57.1% of the sample were retired.

### 2.4 Data collection

#### 2.4.1 Focus group discussions

Two FGDs with 19 patients (FGD1 = 12; FGD2 = 7) who received the MCI were performed in February and July 2020, respectively, to assess the programme’s socially constructed meaning using an interview guide. The meetings were conducted in the same room and primary care centre. They were audio-recorded, with prior signed informed consent, and the verbatims were systematically transcribed, guaranteeing informants’ anonymity.

The FGDs lasted 90 min each, and they were conducted by the first author of this article (PhD candidate), together with a co-moderator (PhD) and an observer (PhD) who performed field notes. Both moderators have education and training in psychotherapy and researching and are experienced with group techniques, while the observer belongs to the biomedical field. Participants had neither previous knowledge nor contact with the qualitative research team before the FGDs, and they were informed about professionals’ backgrounds at the beginning of the sessions. Moreover, the FGDs were attended by three members of the research team and the recruited informers.

#### 2.4.2 Interview guide

The semi-structured interview guide (Supplementary material 1) included open-ended and follow-up questions covering the following topics: patients’ experiences during the MCI programme and their effectiveness overall perception, the adequacy of the frame elements (timeframe, setting, professionals, beneficiaries, group-based intervention approach) and contents, improvable aspects, barriers and facilitators on the intervention implementation, and the benefits on patients’ QOL in daily life. The interview guide was not pilot-tested with FMS patients but reviewed exhaustively by the research team.

### 2.5 Ethical considerations

Helsinki/Tokyo Declaration was followed for the study design being approved by the Clinical Research Ethics Committee of the Fundació Institut Universitari per a la Recerca a l’Atenció Primària de Salut Jordi Gol i Gurina (IDIAPJGol), on 25/04/2018 (code P18/068). Participants received oral and writing information, guaranteeing data protection and anonymity before signing the informed consent sheet.

### 2.6 Data analysis

A thematic analysis (Braun & Clarke, 2006) of the participants’ accounts was carried out to detect outstanding topics through the interpretative methodology. The text corpus and the observer’s field notes were read several times and interpreted by the three qualitative research team members, including preliminary analytical intuitions, a coding process, and the data organization into categories. Saturation of the data was assumed once no more codes could be captured from the data and no more meaningful insights were deduced from them according to the study goals and after an interactive and reflective process of analysis.

Subsequently, the analysts’ triangulation was performed to detect the essential attributes of the narratives, compare perspectives, and reach an agreement on the most prominent domains and emerging themes of the FGDs. Discrepancies were resolved through consensus after reviewing transcripts. As a result, the selected themes were identified and clustered, and an explanatory framework was drafted.

No specific qualitative analyst software was implemented to carry out the coding. Finally, no informers’ feedback was requested for the analysis process.

### 2.7 Rigour

In attendance of the key component of data trustworthiness (Korstjens & Moser, 2018), this study presents two analysts’ triangulations with three research team members compiling credibility. Nevertheless, transcriptions were not returned to participants for comment and correction but double-checked by the research team.

Moreover, purposive sampling has been conducted, including thick descriptions with individual and contextual factors, in order to obtain maximum discursive variability and FMS patient representation for outcome transferability. Even though no external audit trail has been performed to ensure dependability, the research path was documented and described throughout the study section. For instance, the same interview guide was implemented in both FGDs, and three different analysts’ codings were contrasted during triangulation to guarantee the study’s consistency. Additionally, verbatims were transcribed by an external specialist and reviewed by the research team.

Furthermore, confirmability has been achieved by using literal quotations and including researchers’ backgrounds. Finally, preliminary analytical intuitions focused on reflexivity during the thematic analysis to detect possible sources of the investigator’s bias.

## 3 Findings

The thematic analysis results were systematically organized in five domains according to the interview guide with the view of focusing on the purpose of this study and including one central theme that emerged from the answers reported in the FGDs. Additionally, representative quotations are cited to support the interpretation of the corpus text. For this purpose, quotations were translated from Catalan and Spanish to English. Unfortunately, due to some of the participants refused to be filmed, no video-recorded was carried out. Consequently, it was not possible to identify the informers during the transcriptions so that they are presented by FGD instead.

### 3.1 Domain 1: user satisfaction

Theme 1: “A positive but improvable experience”.

The MCI was unanimously embraced by users from both FGDs, describing it as an enriching and positive experience in terms of acceptability. The programme was acknowledged for its educational and health benefits as well as for its positive group cohesion effect.

“I have learned a lot from this programme. I have been with FMS for 19 years now, but I have learned many things about physical activity, eating, about … about many, many things […] Really good; excellent. I feel, let’s see … a little relieved. Anybody can stop your pain but provides you psychological relief. As it is said that this syndrome is just in our heads. Let’s see … when you know that people are feeling worse than you … you know? Be aware of it relieves you. […] From my point of view, it encourages you to live because as the lady said: the family does not understand fibromyalgia, and we are not understood either. The person who has it does understand though. We support each other.” (FGD2)

“I wanted to say that, for me, this whole experience has been incredibly positive, especially emotionally.” (FGD2)

Participants´ acceptance is strongly related to their feeling of being acknowledged, understood, comforted, and accompanied, especially by peers:

“Discovering that you are not alone.” (FGD1)

“To have someone who is going through the same as you, who understands you completely.” (FGD2)

The identification process involved, given by the group cohesion effect, confirmed their illness, validating them as legitimate patients from a legitimate health condition. Thus, FMS legitimacy, not only as a diagnostic category but as a frequent and genuine affliction, represents one of the most critical informants’ concerns:

“How can I demonstrate that I have this pain? […] I cannot prove it scientifically.” (FGD2)

“I think you made an excellent programme. It reinforced something very important, which is accepting and assuming the diseases […]. I have learned more from my colleagues than from the doctor. Why? Because each experience is unique, and each case is singular.” (FGD2)

The group involves a task, a purpose, and a common objective, finding its participants in an environment of unity and representativeness. Social interaction shapes subjectivity in multiple directions and, as it is expressed in the first quote, “*it encourages you to live”*. Therefore, group cohesion is presented as vital, it represents life drive, and therefore, it embodies well-being and health.

“When the course was over, we used to meet once a week to practice what we had learnt and to spend some time together.” (FGD2)

Overall, informers reported being satisfied with the professionals’ performance. However, participants from the second FGD questioned communication skills, specifically when practising physical activity.

“They worked with goodwill; I can tell. The problem was that they insisted too much on ‘walk, walk, walk’. But what if I do not like so much this type of work out and I prefer something else? I think they do not really understand our limits […]. I think they did not adjust their methods to our condition. However, it was not deliberately, I believe […].” (FGD2)

“We would rather be motivated more softly.” (FGD2)

“We are very susceptible. Generally, nobody listens to you, nobody understands you, and on top of that, professionals put pressure on you. Consequently, you end up in a terrible mood. Truly. We are highly emotionally sensitive people, easily overwhelmed. You do what you can, but If you add a burden to this sensitivity, the body just cannot handle it.” (FGD2)

The proposed MCI was also considered feasible to be incorporated into usual clinical care, replicable in other health contexts, and recommended to other patients with FMS by the informers.

“Would be useful to get prompt access to therapy service and receive psychological support more frequently.” (FGD1)

“The programme has gone very well, but it should not end here … it should be a continuum, […] we should have regular psychological help.” (FGD2)

### 3.2 Domain 2: users’ overall perception of MCI effectiveness

Theme 2: “Health improvements and suffering relief”.

Regarding the perceived health benefits, informers from both FGDs agreed in the positive impact of the MCI on their QOL. Nevertheless, they do not report significant changes in physical symptoms such as pain. On the other hand, they explained that the MCI allowed them to improve their lifestyles by incorporating healthy habits and routines, reducing the pharmacological intake, developing a positive attitude towards pain, and enriching psychological and social well-being.

“I do think that we made progress with our quality of life because we had the opportunity to relate to others with the same problem and who understood our suffering. […]. Nevertheless, it is not so simple as it looks. Even though you can ‘look better’ for the rest of the world, any extra physical effort could leave you motionless for days.” (FGD2)

“We were taught about the different symptoms, the pain, the consequences of medication … but we need to be aware of what we are going through, how we feel, and what our limits are. […]. If we know our needs and limits in-depth, we can avoid complaining and self-compassion. […] Doing so, I have managed to lower the medication by half being proactive and energetic, even not feeling well.” (FGD1)

“I am in pain from the moment I get up. I know that the pain is not going to disappear, but I try to avoid it somehow. So, I keep taking my medication, and I try to take short breaks and rest if I feel tired. I also practice physical activity regularly. I started swimming, and I go to the sauna and the jacuzzi. Honestly, I am doing very well now. I try to do it every day for at least one hour.” (FGD1)

Additionally, participants considered the MCI as an eye-opening experience given the alternative non-pharmacological treatment strategy offered.

“Participating has helped me out to enhance as I had the chance to get what I needed. And then, when it finished, I knew what another kind of healthcare I had to look for. In other words, this programme opened new doors for me.” (FGD1)

This experience’s benefits have proved to go beyond the symptomatic relief of FMS, offering patients a new perspective on the health-disease process. The informers emphasized the acquisition of self-understanding, self-control, and self-management of the syndrome.

“In my case, I have noticed self-help improvement, learn how to help myself […] I have managed to get to know my problem so deeply that now I can say to FMS: “I am the boss, not you”. Do I still have pain? Absolutely […] But even so, I have managed to reduce my medication which means a better health status. […] First me, then me, and always me. Here is my conclusion.” (FGD1)

“You cannot get rid of the pain, but you can learn how to control it.” (FGD2)

Patients realized that FMS’s psychological implications do not make it less real or tangible in their daily suffering physically, emotionally, and socially. Indeed, informers accepted that psychological well-being is essential to symptom management, as experienced during the intervention programme.

“The doctors told me that I had nothing. So, what can you do with that answer? Even though all your body hurts, you do not know where to go … I was told it was all psychological. But no matter how psychological it can be, it hurts me … ” (FGD2)

“I have been thinking about all the medication that I am taking, and I would like to cut it all down as I found that this condition is more about your emotions … I have experienced that when I have problems, or I feel upset, I feel physically worse […] So, I believe that treating our psychological needs can actually help us more than any medication.” (FGD1)

In resonance with the mentioned benefits, this intervention programme has contributed to awakening participants about gender disparities in health and the socially constructed roles of men and women.

“I firmly believe that as women, we have much more burden than men to cope with all kinds of situations in life. […] It is evident for me that women make much more effort in daily life than men.” (FGD1)

“Currently, women relate more and more to each other. And from these exchanges, you can conclude that we are the real family pillar [.]. I spent my whole life taking care of others […]. So then you understand that we carry a burden that is very difficult to deal with. I am not surprised what is happening to us considering the accumulated stress in our bodies.” (FGD1)

“The problem of our generation is that we were taught to shut up. Around my 40ish, a woman told me once: ‘you were born in the time of the mutes’. I was astonished. Then she asked me: ‘Have you ever answered your parents unproperly?’ ‘No, and I am also very cautious with my children’, I said. ‘Now you see that you belong to the voiceless times?’, she replied. […] But when you get to a certain age, your temper comes out like a boiling pot, and no one will ever be able to stop it. Once it arises, no one can do anything about it anymore.” (FGD2)

### 3.3 Domain 3: users’ opinions about the MCI format and framework

Theme 3: “Correct but limited”.

Informers from both FGDs claimed that the programme timeframe was not enough to cover in-depth all its contents and offer them time to work on their suffering.

“It was insufficient … Not for professionals’ quality. […]. Each participant has her/his needs, and there was not enough time to dedicate to everyone. That is why it was not enough.” (FGD1)

“The problem was the lack of time to delve a little deeper into the different topics. Moreover, because each person had things to say and issues to share, and there was not enough time for everybody.” (FGD1)

“I believe that a little more quantity would guarantee more quality. Extending the timetable will provide better quality over time.” (FGD2)

Regarding the group approach, informers showed acceptance, acknowledging its benefits but also remarking its drawbacks.

“In my opinion, the group approach has been very helpful. We have been incredibly brave dealing with this condition every day, this backpack that … and we needed to learn to get rid of things that we carry inside the backpack. It was very important and beneficial as we could identify ourselves with our colleagues’ suffering so that I no longer felt alone again.” (FGD2)

“The problem with large groups is that … if all the members of the group talk, then you are suddenly run out of time.” (FGD2)

“But the good thing with large groups is that you can find plenty of different scenarios. Some people have fibromyalgia terribly, and some others manage it better. Some people already accepted this condition, and some others are still in process. Therefore, you can learn about each experience and keep what better suits you.” (FGD2)

“In my case, I have many health issues. Therefore, listening to my partners’ problems make it worse for me. […] Sometimes, the group could make me feel overwhelmed due to the emotional burden it entails.” (FGD1)

Additionally, participants proposed including the family and general practitioners in the sessions in order to inform them about the characteristics of this condition, the related suffering, and patients’ real health needs.

“I do not know how this could be managed to inform family members about our suffering and what we are going through. Maybe they should be invited to a session […]. We need them to be aware of the consequences of this condition and our daily fight to cope with it.” (FGD1)

“I think that a family member should attend the meetings, and especially those who are incredulous so that they comprehend why it hurts so much even when you do nothing. They usually say: ‘If you are doing nothing, you should not feel pain.’ And it makes sense that they cannot find it a logic, but sometimes it can be tough for us to explain it adequately.” (FGD2)

“To include general practitioners into the sessions to make them comprehend the problem of their patients […]. I was diagnosed recently, but I have been twelve years with this pain visiting several doctors without an accurate response. That is not normal.” (FGD2)

### 3.4 Domain 4: users’ opinions about the MCI thematic content

Theme 4: “Relevant topics but psychological support should be reinforced”.

Towards health education, sexuality was mentioned as a relevant theme briefly explained during the intervention programme. Informers from the first FGD showed concern about coping with FMS in their sexual life and communication with their sexual partners. Indeed, the interpretative analysis revealed participants´ veiled worry about the side effects of medication on sexual libido.

“When we talked about sexuality, it was all speedy, inhibited and shyly. We could not speak freely about it. For example, in my case, my husband does not understand that I do not feel like having sex lately […]. But I am the one who has the problem, it’s me … I just can’t because I am over medicated, I am nervous, I cannot stand to be touched … and he cannot comprehend me. We are in serious trouble. I think these issues should be addressed properly in the programme, among other things. Sexuality was a subject that we saw very quickly, very quickly, very quickly, as well as the medication matter […].” (FGD1)

“When you do not know how to explain to him why you feel bad … sexually. But we should make this clear to the people we live with. Sometimes, it is not because we do not want to; it is just because we cannot. And this is something very complicated to be understood and be explained.” (FGD1)

Accordingly, there is a scope for further clarification about pharmacological treatment. Informers expressed doubts and confusion regarding its efficacy, specificity, side effects, and administration. Furthermore, a trend towards self-medication and self-administer treatment without professional guidance was observed. Besides, participants expressed disbelief about the effectiveness of the pharmacological approach.

“The presentation of this issue was very light and superficial, and we ended up with a ton of doubts.” (FGD2)

“We lack information about medication because they just talked about it in general during the course, and then a little bit about anxiolytics and antidepressant.” (FGD1)

“I have been taking pills for almost 19 years, and now I am developing a drug allergy. I honestly I feel worse and worse. I wish there were something more natural.” (FGD2)

Participants from the first FGD also suggested improving memory and nutrition sessions since they found them shallow.

“Another topic that has not been properly discussed during the programme is memory.” (FGD1)

“In my opinion nutrition is essential, but unfortunately, we did not get any special diet or food guidelines from this course.” (FGD1)

As mentioned throughout the results, informers emphasized that the psychological component is crucial when living with FMS and demanded regular access to this type of treatment as part of the UCC. Overall, participants highlighted the coping skills acquired during the programme. Nevertheless, they expressed the need for including a therapy group with a psychodynamic approach to work on their emotional issues and psychological suffering in addition to cognitive-behavioural techniques. The interpretative analysis showed that they also need to be heard and express their feelings and concerns in a therapeutic context and not exclusively educational. According to their experiences, the intervention programme did not provide the appropriate framework for it.

“About the psychology sessions, it would be helpful if you could organize groups of 10–12 people, once a month, and led by a psychologist to work out on mental health issues instead of just give us some guidelines.” (FGD1)

Finally, informers showed resistance to the methodology implemented as it has been stated above regarding physical activity. Even though some participants reported they had incorporated exercise in their routines after the programme, others admitted that it was impossible for them due to the pain and physical limitations.

“Well, the physio recommended us several exercises and stretches, but the thing is … you try to walk and then you cannot move for two days. Even when you try with water gym because … but then I am two or three days that I am motionless.” (FGD1)

“Let’s see, they gave us guidelines, and I do some exercises at home. Well, I do the exercises, and I do not do them … Even if I am tired or for whatever reason, I try to do it anyway. […] And every ten days or so, I also take a physiotherapy session, which is truly helpful.” (FGD1)

### 3.5 Domain 5: additional users’ improvement proposals

Theme 5: “Supporting material reorganization, social backing and prevention strategies”.

Regarding the supporting materials, informers proposed delivering it in dossier format instead of giving single sheets at the beginning of the programme, to avoid losing relevant information and not attending a session.

“Today I give you this sheet; tomorrow I will give you that one; the day after tomorrow, this other one and so on. Suddenly you realized that you had missed a paper someday or you had lost it somewhere.[…] I think they should organize it better, at least delivering this material all together by areas such as the gymnastic guideline, the psychological guideline, etc. […] Otherwise, we had to ask our colleagues for the missing sheets.” (FGD2)

Another emerging theme was the need for social support, in terms of access for a financial benefit specifically due to fibromyalgia diagnosis, for those unable to continue working actively or require early retirement.

“For example, I would like that if you could support with Fibromyalgia literacy to members of the medical board. Many people suffering from this disease do not get any social benefit from it. In my case, I do not have a pension, but I heard about people who do. But just a few. The majority of us do not have access to it.” (FGD2)

Finally, it was suggested to include prevention strategies to avoid health status deterioration and personal autonomy loss in the long term.

“I am very concerned about the future. […] I would like to be more autonomous as I get old, but of course, I don’t want that … I don’t want to depend on anyone as much as possible. But about this point, I do think that the programme could work on preventing deterioration. Trying to ‘anticipate’ what awaits us and work out on acceptance […]” (FGD1)

## 4 Discussion

This study presents patients’ accounts about an MCI programme for FMS conducted in Catalonia´s primary care settings. Based on this valuable information, adjustments were identified to adapt the intervention and increase its benefits. The results suggest that the informants’ expectations have been fulfilled thanks to the proposed multidisciplinary and comprehensive treatment approach, allowing them to bond with people under the same health condition. Consistently with another study, the FGDs revealed patients’ dissatisfaction with the UCC and the need for more effective and less harmful alternative strategies (Briones-Vozmediano et al., 2013).

Even though FMS diagnosis may first concede hope to patients, it becomes an empty promise, as Boulton (2019) describes, since neither provides a final solution nor social legitimacy. Given the lack of a diagnostic test, peers’ pain becomes a relief working as a mirror, reflecting belonging and support, and representing living proof of FMS authenticity. The more a peer suffers, the more FMS is proved, as said in the first informant’s quote. The group re-signifies the individual suffering transforming it into a meaningful and valuable experience. Indeed, the group myth arises as an account for what cannot be medically explained. It is a narrative construction that gives meaning to an inexplicable phenomenon closing a knowledge gap, raising a structure, and defining the group’s history and path’s edges. In other words, the group effect consists of finding something familiar from something unknown, conferring a relieving and comforting explanation that is better than none (Nietzsche, 2003). Therefore, the satisfaction to the proposed MCI seems to lay in the symbolic efficacy (Levi-Strauss, 1978) of a socially constructed and shared myth about FMS. Our results are in general agreement with Oliveira et al. (2019), on the repercussions of interdisciplinary intervention for FMS women, about the group’s positive influence on changing health habits and behaviours and its psychological support benefits. Furthermore, another study suggests that offering patients with alternative narratives could benefit their self-perception and lifestyle change (Hyland et al., 2016). Consequently, our results confirm that the group cohesion represented, in most cases, a facilitator to the MCI implementation, which should be considered for future treatment designs.

While expecting an improvement in symptomatology, the MCI proved to cover a wide range of health needs beyond physical indicators, as Bourgault et al. (2015) observed. Accordingly, participants adopted a new active role in their illness experience that increased autonomy and empowerment. In addition, recognizing FMS frequency between peers played a key role in patients accepting this disorder, prioritizing and addressing their health needs. Hence, this initial insight entailed the first step in their health processes. This finding mirrors a recent study (Tangen et al., 2020), concluding that pain acceptance is associated with better functionality and fewer FMS symptoms.

On the other hand, physical activity benefits for this specific MCI depend partially on participants’ will in exercising afterwards what they have learned during the programme. Should not implement participants self-management recommendations as part of their lifestyle could become a barrier to educational programmes. Thus, informants might not have perceived any significant symptomatic change, particularly pain reduction, as they have probably not practised enough post-intervention. Some of the possible reasons could have been accessibility, lack of social support, activity-induced pain, lack of motivation, among others. However, our results are supported by Merriwether et al. (2018), who observed that lifestyle physical activity is positively associated with function and fatigue but not pain.

Nevertheless, considering FMS patients’ tendency to emotional lability and low pain tolerance threshold, it seems necessary to promote their enthusiasm and commitment through reviewed tailored tactics respecting their pace and encouraging physical work. A previous study (Larsson et al., 2020) reported that adjusting exercises and the pace, avoiding overload, offering enjoyable activities, and creating the right conditions were crucial factors when promoting physical activity. Future adjustments to the proposed MCI should therefore consider incorporating a person-centred rehabilitation approach and adding extra physical training sessions to ensure a minimum of continued and supervised exercise.

Overall, our results provide compelling qualitative evidence for educational programmes oriented to acquiring multiple skills to cope with FMS. The presented MCI has not been designed to replace the UCC but to reinforce it providing patients with a more holistic approach, including multidisciplinary and non-pharmacological methodologies. Participants reported having been taught several thematic contents and strategies to reduce FMS impact on their QOL. Nevertheless, an expressive psychotherapy approach has been suggested to help patients face psychological suffering and prevent voiceless, especially considering the total pain experience registered, which involves physical, spiritual, psychological, and social suffering (Williams & Craig, 2016). For instance, Lumley et al. () designed emotional awareness and expression therapy, showing promising results compared to CBT.

Informants described FMS pain as disruptive, unpredictable, and ungovernable, interfering with daily life and interpersonal relationships. These findings extend those of Ashe et al. (2017), confirming FMS’s biopsychosocial impact. Pain suffering entails a subjective experience but subjectivizing insofar as it challenges all the individual’s spheres. Total pain copes with the body as a whole where, according to informants’ words, “everything hurts” without boundaries or gradient. As a result, they feel frustrated when distinguishing FMS symptoms from other comorbidities. This feeling of hopelessness could be accountable, among other aspects, for patients’ seeking disability health insurance, which excludes them from the social production system.

However, a health programme should encourage patients to rejoin the social dynamics and perform active roles. From this perspective, including relatives in the programme could promote healthier family relationships based on comprehension and destigmatization. Alameda Cuesta et al. (2021) explain that the ultimate aim of healthcare for FMS patients should be to decrease vulnerability and exclusion. A family approach would also allow implementing a gender perspective working on gender roles in the family group. Additionally, inviting reliable sexual partners into the programme could help patients cope with sexual dysfunction (Granero-Molina et al., 2018). According to the evidence, one of the side effects of antidepressants, commonly prescribed for FMS, is serotonin overproduction associated with libido inhibition (Lorenz et al., 2019). Consequently, sexual climax could be seriously disturbed, which could frustrate pleasure and compromise couples’ sexual life and relationships (Matarín Jiménez et al., 2017; Romero-Alcalá et al., 2019).

The present study contributes to detecting barriers and facilitators for implementing an MCI according to participants’ appraisals. Even though this programme must be tailored based on detected weaknesses in format and content, it offers a promising approach to be incorporated in the UCC.

### 4.1 Limitations

In reference to the methodological limitations, these study results are not generalizable, although the intervention programme can be considered a model experience replicable in other contexts. Attending the nature of self-reported data, group effect, memory, selection, or attribution biases could have affected the results.

In addition to the method shortcomings, any preliminary qualitative implementation study was not conducted for the proposed MCI. Moreover, the FGDs sample only included those who consented to participate in the qualitative study and had a high assistance record; therefore, overestimation bias could have been possible. Besides, the sample has no male representation due to women’s FMS hight-prevalence, and since the only two possible men candidates did not accept participating in the qualitative study. Therefore, they were no focus groups according to sex.

Furthermore, the sample includes patients from only 7 of the 11 primary care centres of the region as the MCI groups performed in the rest of the centres did not meet the time inclusion criteria (a maximum of 12 months of follow-up). Additionally, participants were Spanish and except only for one informant. Even though two informants did not fulfil the attendance inclusion criteria (presenting only 66.7%), they were recruited to join in the FGDs to compensate for possible no-shows and guaranteeing sample variability. Finally, FGDs differ in the number of participants due to the eligible sample availability by follow-up criteria and the COVID-19 outbreak, which impacted the recruitment process of the second FGD.

Regarding the data collection, no video recording was performed, and, consequently, the quotations could not be presented identified by the informer but by FGD. Lastly, no external expert was included in the analytics triangulation processes since discrepancies were solved by consensus.

### 4.2 Strengths

Among the strengths, this study has been drafted based on the literature and according to the consolidated criteria for reporting qualitative research (COREQ) (Tong et al., 2007) and the standards for reporting qualitative research (SRQR) (O’Brien et al., 2014). Implementation research guidelines have also been considered (Brownson et al., 2012; Proctor et al., 2009). Moreover, the recruitment strategy and the sample description were performed according to high-research standards to enrich variability and avoid biases. For the analysis process, two analytics triangulations were conducted involving three experienced investigators. Methodologically, based on critical thinking, a hermeneutic interpretative analysis was implemented to assess the narratives in-depth. Finally, this study’s results provide a depth comprehension of patients’ live experience with FMS treatment, strengthening primary care professionals’ understanding and daily practice.

## 5 Conclusion

In summary, no significant contradictions were observed between the study groups in the thematic analysis results. Both FGDs expressed a positive experience with the MCI programme, mainly related to the group cohesion effect. The global perception of effectiveness indicates no substantial differences between short- and long-term health benefits. Informants perceived an improvement in their QOL and highlighted the benefits of emotional, psychological, and social levels over the physical-symptomatic control. Additionally, other health benefits were registered, such as reducing the medication, acquiring a healthier lifestyle, awareness of their health needs and self-care, autonomy, and gender awareness. Therefore, the MCI fulfilled users’ overall expectations about results and procedure.

In closing, the informants accepted and valued the MCI due to its holistic perspective, safety, health benefits, and FMS legitimacy promotion. Although identified adjustments should be performed to cover patients’ real health needs, the proposed programme accomplished its main goal according to participants’ appraisals requesting its continuation as part of the UCC.

## Supplementary Material

Supplemental Material

## Clinical Trial registration number

This qualitative study is linked to the clinical trial number: NCT04049006 (ClinicalTrials.gov)
