# Supplementary material for: Patients’ appraisals about a multicomponent intervention for fibromyalgia syndrome in primary care: a focus group study
Source: Int J Qual Stud Health Well-being. 2021 Nov 29;16(1):2005760. doi: 10.1080/17482631.2021.2005760 (PMC8843386; doi:10.1080/17482631.2021.2005760)
Supplement: Supplemental Material [file ZQHW_A_2005760_SM2783.docx]

**SUPPLEMENTARY MATERIAL 1**

Focus Group Discussions Interview guide

1. Greetings and presentation of the research team.

2. General information on the focus group discussion (FGD).

3. Explanation of the ethical and confidentiality aspects.

4. Explanation of the FGD dynamics.

5. Individual presentation of the informants.

6. FGD questions:

1. What do you think as a whole about the group programme for Fibromyalgia?
2. To what extent have you found it helpful?
3. What have you liked the most about the programme?
4. What have you liked the least?
5. What aspects of the programme do you think could be improved?
6. How have you felt participating in a group programme compared to the individual clinical care you usually receive?
7. Regarding the programme´s setting features, what do you think of the place and timeframe of the programme?
8. To what extent has this programme helped you to improve symptoms management? For example, have you noticed any enhancement in your Fibromyalgia?
9. To what extent have you noticed any improvement in other aspects of your daily life after participating in the programme?
10. How would you describe the professionals’ performances during the programme?
11. Would you like this programme to be included as part of the usual treatment in primary care centres? Why/Why not?
12. Would you recommend this intervention programme to other Fibromyalgia patients? Why?/ Why not?
13. Would you like to comment on something else?

7. Ending of the FGD:

• Summary of the informants ‘contributions.

• Acknowledgement and thanks.
